# Marine ice regulates the future stability of a large Antarctic ice shelf

**DOI:** 10.1038/ncomms4707

**Published:** 2014-04-22

**Authors:** Bernd Kulessa, Daniela Jansen, Adrian J. Luckman, Edward C. King, Peter R. Sammonds

**Affiliations:** 1 Glaciology Group, College of Science, Swansea University, Singleton Park, Swansea SA2 8PP, UK; 2 British Antarctic Survey, Natural Environment Research Council, Madingley Road, Cambridge CB3 0ET, UK; 3 Rock and Ice Physics Laboratory and Centre for Polar Observation and Modelling, Department of Earth Sciences, University College London, Gower Street, London WC1E 6BT, UK; 4 Present address: Division of Glaciology, Alfred-Wegener Institute for Polar and Marine Research, 27568 Bremerhaven, Germany

## Abstract

The collapses of the Larsen A and B ice shelves on the Antarctic Peninsula in 1995 and 2002 confirm the impact of southward-propagating climate warming in this region. Recent mass and dynamic changes of Larsen B’s southern neighbour Larsen C, the fourth largest ice shelf in Antarctica, may herald a similar instability. Here, using a validated ice-shelf model run in diagnostic mode, constrained by satellite and *in situ* geophysical data, we identify the nature of this potential instability. We demonstrate that the present-day spatial distribution and orientation of the principal stresses within Larsen C ice shelf are akin to those within pre-collapse Larsen B. When Larsen B’s stabilizing frontal portion was lost in 1995, the unstable remaining shelf accelerated, crumbled and ultimately collapsed. We hypothesize that Larsen C ice shelf may suffer a similar fate if it were not stabilized by warm and mechanically soft marine ice, entrained within narrow suture zones.

## Abstract (web summary)

Signs of instability in the Antarctic Larsen C ice shelf have raised concerns that it might soon collapse like its northern neighbour Larsen B. Kulessa *et al.* combine an ice-shelf model with satellite and geophysical data to show that despite dynamic similarities, Larsen C is presently stabilized by marine ice.

## Graphical abstract

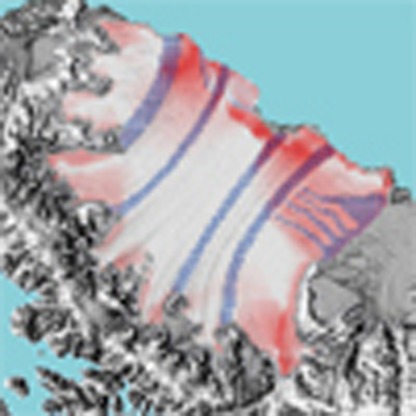

The stability of an Antarctic ice shelf depends on the balance between the constructive stresses that ensure its integrity and the destructive stresses that compromise it. The spatial distribution and temporal evolution of the first and second principal stresses on Antarctic ice shelves depend on the geometry of the embayment in which the shelf is situated, the spatial and temporal attributes of the ice recharge it receives from feeding glaciers, environmental factors such as the evolution of atmospheric surface and oceanographic basal conditions^1^, and the physical properties of the types of ice of which it is composed^2^,^3^. On many Antarctic ice shelves, including Larsen C (Figs 1 and 2), tensile stresses dominate over compressional stresses. The normal stress with the highest magnitude, the first principal stress, therefore commonly controls the magnitude and direction of local strain rate and ice-shelf extension, and by implication in uniform strain-rate fields therefore the direction of extensional flow.

Fractures, such as horizontally extending rifts and vertically and horizontally extending surface and basal crevasses can be inherited from feeding glaciers. Fractures may also originate near the grounding line as the ice begins to float, or open up on the shelf in response to bending or shearing forces or thermal processes^4^,^5^. Such fractures commonly strike orthogonal to ice flow, as typified, for example, on Larsen C ice shelf where several cross-flow zones of major and minor fracturing are present (Fig. 1). Within such zones, rifts and crevasses often form regular sequences that share the same formative conditions^3^, such as, for example, the major rift sequences ‘R1’ to ‘R5’ (Fig. 1). Only on rare occasions do fractures intersect the ice-shelf’s flow lines at oblique angles, such as in the downflow sectors of sequences R1 and R5 (Fig. 1). If the tensile first principal stress is aligned perpendicular (parallel) to a fracture’s strike then that fracture’s opening rate is maximized (minimized). As fractures tend to strike perpendicular to flow, we propose that the shelf-wide distribution of the angles between the flow direction and the first principal stress (hereafter ‘stress-flow angles’) can serve as a first-order criterion on which to judge an ice-shelf’s stability. Fundamental to a quantitative prediction of Larsen C’s stability in a warming climate is therefore the assessment of the distribution and climatic sensitivity of the stress-flow angles across the ice shelf, and it is this factor that we address in this manuscript.

We adopt our previous approach^4^ and model ice-shelf velocities, flow lines and stresses with a continuum-mechanical ice-flow model with spatially uniform ice properties. In subsequent sensitivity studies, we ascertain the impact of spatially variable ice properties and changing model boundary conditions on modelled stress fields and stress-flow angles. More specifically, we follow a three-stage line of argument to demonstrate that marine ice stabilizes Larsen C ice shelf. First, the distribution of stress-flow angles across Larsen C ice shelf is analysed to demonstrate that its frontal portion provides critical restraint for the otherwise unstable central portion. Second, we infer that the rapid disintegration of Larsen B ice shelf between 1995 and 2002 might serve as a plausible blueprint for Larsen C’s future demise, because Larsen B was characterized by an equivalent distribution of stress-flow angles pre-collapse. Following a brief introduction to marine ice-bearing suture zones and their anomalous physical properties, we argue, third, that such zones presently prevent the loss of Larsen C’s frontal portion and by implication, therefore, stabilize the whole ice shelf.

## Results

### Stress-flow angles on Larsen C ice shelf

**Figure 1:**
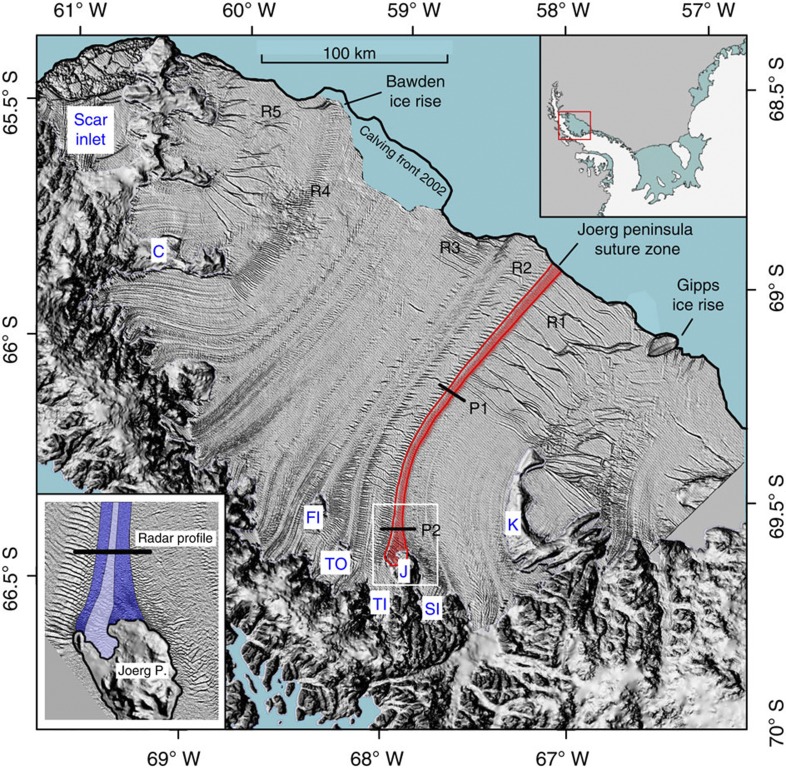
Larsen C model domain. Aster-Global Digital Elevation Map (GDEM)-( http://nsidc.org/data/docs/agdc/nsidc0516-cook/) derived Digital Elevation Model (DEM) is superposed on a 2008 Moderate Resolution Imaging Spectroradiometer (MODIS) image ( https://earthdata.nasa.gov/data/near-real-time-data/rapid-response). Top inset shows the location of Larsen C ice shelf on the Antarctic Peninsula. The red stripe in the main figure traces the Joerg Peninsula (J)-derived suture zone. P1 and P2 mark the locations of the two ground-penetrating radar profiles shown in Fig. 4. The Joerg Peninsula, Tonkin Island (TO), Francis Island (FI) and Churchill Peninsula (C)-derived suture zones separate prominent areas of rifting (‘R1’ through ‘R5’; part of the Churchill Peninsula (C)-derived suture zone southwest of label ‘R4’ is obscured by clouds). The white box in the main figure outlines the location of the bottom inset. In this inset, the dark blue stripe overlain on a high-resolution Landsat image marks the area of marine-ice accretion. The newly formed suture zone becomes laterally compacted by the neighbouring converging Trail-Inlet (TI) and Solberg-Inlet (SI) flow units. The lighter purple stripe enclosed by the dark blue stripes traces the meteoric ice contribution from a small glacier on the Joerg Peninsula, as revealed by our ground-penetrating radar data (Fig. 4).

**Figure 2:**
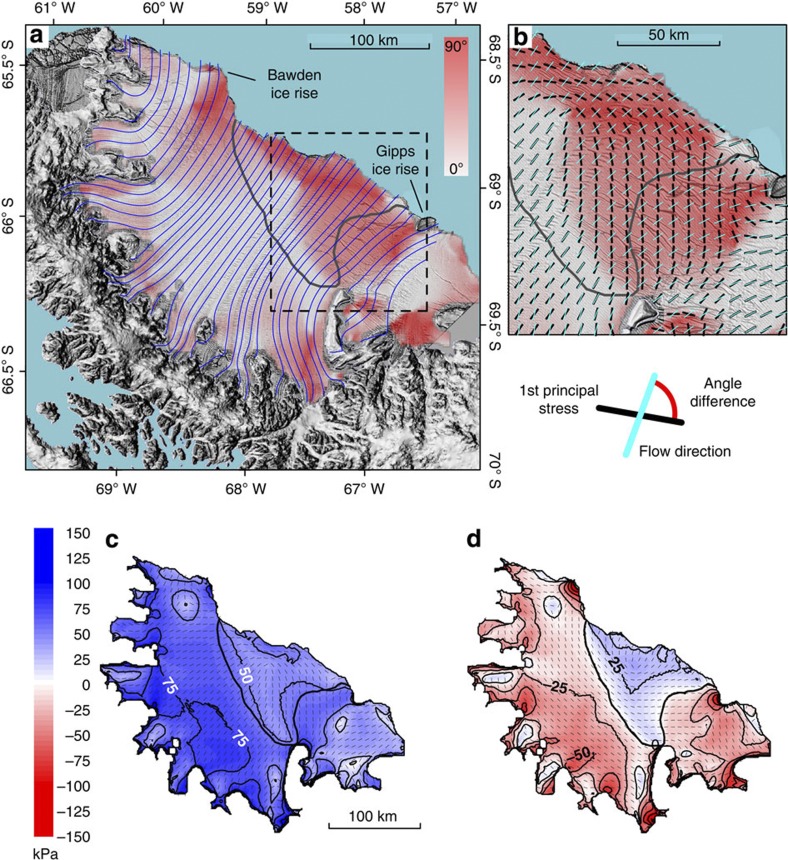
Principal stress fields and stress-flow angles on Larsen C ice shelf calculated from modelled flow-velocity data. Aster-GDEM-derived DEM ( http://nsidc.org/data/docs/agdc/nsidc0516-cook/) is superposed on a 2008 MODIS image ( https://earthdata.nasa.gov/data/near-real-time-data/rapid-response). In all images, the compressive arch is illustrated by a thick solid line. (**a**) Stress-flow angle distribution for the whole Larsen C ice shelf. Part of the Churchill Peninsula-derived suture zone is obscured by clouds. Light blue lines illustrate the ice-shelf’s modelled flow lines, and the dashed box outlines the close-up shown in **b**. (**b**) Concept and close-up of stress-flow angles in Larsen C’s south-eastern sector. (**c**) Magnitude (colours) and direction (grey dashes) of first principal stresses. (**d**) Magnitude (colours) and direction (grey dashes) of second principal stresses.

Our first stage of argument considers that both the first and the second principal stresses have highest absolute magnitudes near Larsen C’s grounding line and decrease non-uniformly towards the calving front (Fig. 2). Stress-flow angles tend towards 90° near Larsen C’s grounding line (Fig. 2a) where feeding glaciers accelerate and spread out laterally as the ice begins to float and basal drag is removed. In the central portion of the ice shelf, the first principal stress aligns with ice-shelf flow (Fig. 2c), and the stress-flow angles thus approach zero (Fig. 2a). Downstream of the embayment, stress-flow angles once more tend towards 90° as the ice shelf spreads out laterally (Fig. 2a,b). Here the tensile first principal stress is oriented along the ice front and parallel to rift zones R1 to R3 (Figs 1 and 2a), favouring a stable ice shelf. Consistently, Larsen C’s calving style is presently dominated by infrequent detachment of large tabular icebergs, as demonstrated by the most recent large calving event in 2008 (Fig. 1). With the removal of the stabilizing frontal portion, the largest tensile stress would be oriented perpendicular to the calving front and to the surface and basal crevasses^5^,^6^,^7^ that are numerous in this area, thus destabilizing them. Larsen C’s frontal portion therefore provides essential restraint for the shelf’s central portion that may otherwise be unstable. The transition from compressive to tensile second principal stresses in Larsen C’s central to southern frontal portion defines a ‘compressive arch’^8^ (Fig. 2d), extending from the Kenyon Peninsula in the south towards the Bawden ice rise in the north (Figs 1 and 2a). It was proposed previously that ice-shelf retreat beyond a critical arch may result in rapid disintegration^8^. Because the transition from low to high stress-flow angles is located closer to the calving front than the compressive arch (Fig. 2a), a hypothetical retreating Larsen C ice shelf may become unstable well before the compressive arch is breached.

### Stress-flow angles on Larsen B ice shelf

**Figure 3:**
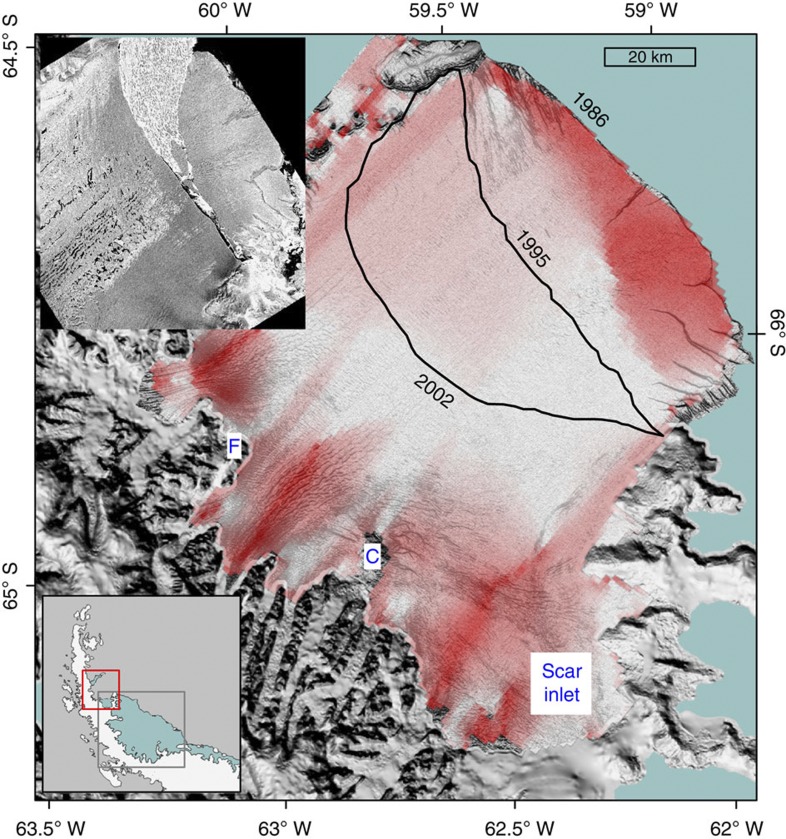
Stress-flow angles on Larsen B ice shelf calculated from modelled velocity data. The background is a Landsat image taken on 1 March 1986 ( http://nsidc.org/data/nsidc-0280.html). The red box outlined in the bottom inset shows the location of the Larsen B ice shelf in relation to the Larsen C ice shelf (grey box outlined) on the Antarctic Peninsula. The 1995 and 2002 calving fronts are shown in thick black lines, and the major calving event that removed Larsen B’s entire frontal portion in 1995 is shown in the European Remote Sensing Satellite (ERS) image in the top inset.

Our second stage of argument considers that the distribution of stress-flow angles on Larsen B ice shelf in 1986 before its collapse (1995–2002, Fig. 3) was similar to those of present-day Larsen C (Fig. 2a), and the stress field in Larsen B’s frontal portion was likewise marked by a compressive arch^8^. Larsen B’s evolution towards rapid disintegration in 2002 (ref. 1) may therefore offer insights into Larsen C’s future stability. Comparable patterns of first principal stresses perpendicular to the flow direction at the grounding line and calving margin, and parallel to flow in the centre, were present before 1995 (ref. 8), and Larsen B calved large tabular icebergs just as Larsen C currently does. In 1995, Larsen B’s entire frontal portion calved away (top inset in Fig. 3), resulting in near-zero stress-flow angles at the new calving front and fracture-orthogonal tensile stresses that then encouraged the propagation of existing rifts and crevasses. Larsen B’s load-bearing capacity subsequently decreased^9^, causing the shelf to crumble by frequent calving of small icebergs and its calving front to continue to retreat (Fig. 3), concluding in its eventual collapse in 2002 (refs 9, 10). Owing to the similarity of shelf-wide stress-angle distributions, Larsen C might similarly disintegrate if its frontal portion, and thus the restraint it provides for the shelf’s central portion, is lost. Ongoing preservation of Larsen C’s frontal portion is therefore necessary for its stability.

### Sensitivity of model outputs to boundary conditions

Larsen C ice shelf has experienced recent mass and dynamic changes that are particularly pronounced in its northern part. These include ice-shelf acceleration^11^, surface lowering due to melt-driven firn compaction^11^,^12^,^13^,^14^ and thinning of feeding glaciers^15^. We have therefore conducted a series of perturbation experiments with our continuum-mechanical ice-flow model to ascertain the sensitivity of Larsen C’s velocity and stress fields to hypothetical changes in the ice-shelf’s calving front geometry, the inflow velocities of the feeding glaciers and the thickness of ice shelf (Supplementary Fig. 1). A comparison of the first and the second principal stress fields before and after a major calving event that occurred between 2002 and 2008 (see superimposed calving front in Fig. 1) demonstrates only weak sensitivity of Larsen C’s principal stress fields to simulated changes in the geometry of its calving front (Supplementary Fig. 1). A 20% acceleration of the feeding glaciers would increase the mean velocity of the ice shelf by 12% from 362 m a^−1^ to 403 m a^−1^. However, because the spatial velocity gradients across Larsen C ice shelf, and thus the strain rates, remain largely unaffected, the first and second principal stress fields have comparable magnitudes and directions with and without acceleration (Supplementary Fig. 1). If Larsen C experienced firn compaction or basal melting that spatially averaged 20 m, its mean velocity would decrease by~7% from 362 m a^−1^ to 338 m a^−1^. However, once again the first and second principal stress fields with and without thinning have comparable magnitudes (Supplementary Fig. 1).

Despite the recent mass and dynamic changes that Larsen C ice shelf has been experiencing^11^,^12^,^13^,^14^,^15^,^16^, our model sensitivity tests thus reveal that the integrity of Larsen C’s stabilizing frontal portion is unlikely to be compromised by mass and dynamic changes in the foreseeable future. Following a brief introduction to ice-shelf suture zones and their anomalous mechanical properties, our third stage of argument considers instead that marine ice-bearing suture zones^2^,^3^ preserve this portion because they prevent rifts from propagating laterally across and coalescing within it; and by implication therefore stabilize the whole of Larsen C ice shelf.

### Marine ice in the Larsen C ice shelf

**Figure 4:**
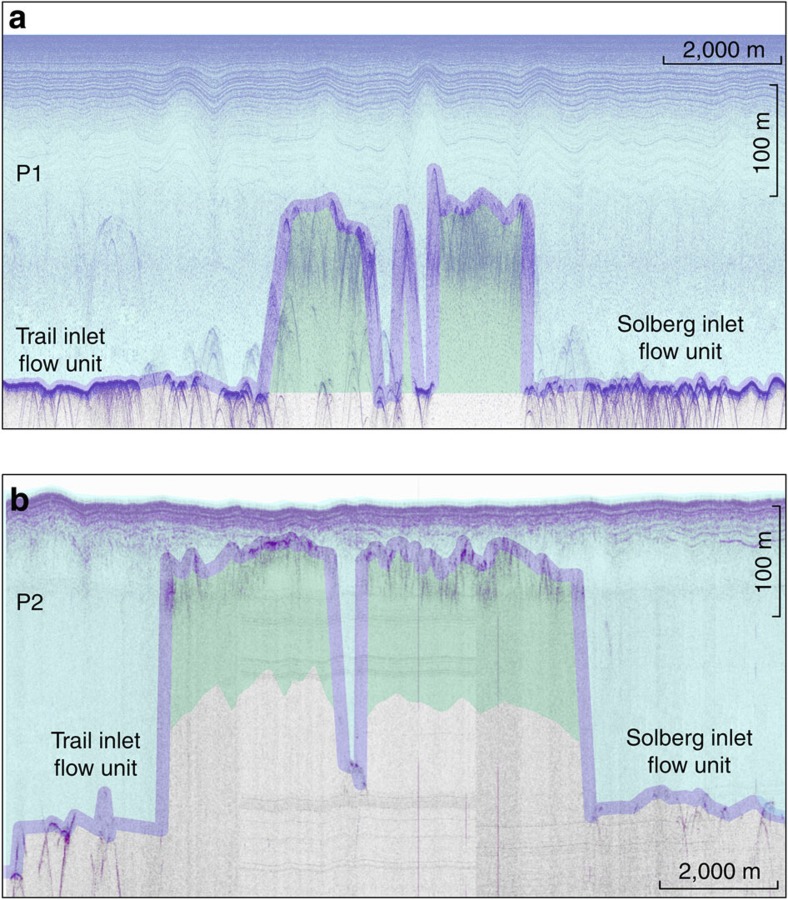
Ground-penetrating radar profiles crossing the Joerg Peninsula suture zone. (**a**) Downstream profile P1. (**b**) Upstream profile P2. The base of the meteoric ice is traced with a purple line, and marine-ice bodies are shown in green. The marine-ice bodies are dissected by meteoric ice derived from the Joerg Peninsula bound glacier (light purple stripe in the bottom inset in Fig. 1).

Larsen C, like most other Antarctic Peninsula ice shelves, is principally composed of flow-parallel units of meteoric ice that are sustained by feeding glaciers and snow accumulation^1^ (Fig. 1), and narrow interstitial suture zones. Suture zones are partially composed of marine ice and commonly appear as smooth flow-parallel bands in satellite imagery^2^ (Fig. 1). Prominent suture zones on Larsen C include those originating leeward of the Joerg Peninsula (‘J’ and red stripe in Fig. 1), Tonkin and Francis Islands (respectively ‘TO’ and ‘FI’ in Fig. 1) in the south, and Churchill Peninsula (‘C’ in Fig. 1) in the north. These zones serve to isolate the prominent areas of fracturing (‘R1’ to ‘R5’ in Fig. 1) in the ice-shelf’s frontal portion. The presence of marine ice within these suture zones was revealed by airborne radio-echo sounding, substantiated by mathematical modelling of sub-shelf freeze-on (ref. 2) and, within Joerg Peninsula suture zone, delineated at high spatial resolution by our ground-penetrating radar surveys (GPR) undertaken in the 2008/09 and 2009/10 austral summers (Fig. 4). Our GPR profiles delineate the base of the meteoric Trail-Inlet and Solberg-Inlet flow units, but cannot detect the suture zone’s base^2^ (Fig. 4a,b). We therefore used seismic reflection data acquired at P1 (ref. 4) (2008–09; Fig. 4a) and calculations of ice-shelf draft at P2 (refs 4, 12) (2009–10; Fig. 4b) to delineate the base of the marine ice within the Joerg Peninsula suture zone. Marine-ice bodies have a temperature similar to the sub-shelf ocean waters from which they are formed (−1.5 °C to −2 °C), and are therefore anomalously soft^17^. In contrast, meteoric ice-shelf units are much colder because they are derived from feeding glaciers and snow accumulation subject to annually averaged surface temperatures of −15 °C and below. Because the thermal diffusivity of ice is very small, the marked contrast in meteoric versus marine ice temperatures is expected to persist along the entire length of an ice shelf^18^. Warmer marine ice deforms more readily under the same long-term stress loading, imposed upon the ice shelf by gravity-driven flow, than colder meteoric ice^12^,^19^. A larger proportion of that long-term loading is therefore available to drive elastic fracture in meteoric than in marine ice, so that warmer marine ice-bearing suture zones are less prone to elastic fracture than surrounding, colder meteoric ice units^20^,^21^.

### Stress-flow angles from observed data

Interferometric Synthetic Aperture Radar (InSAR)-derived flow velocities of Larsen C ice shelf^22^ allow, initially, calculation of the spatial distributions of ice-shelf strain rates. Subsequently, the principal stresses and stress-flow angles at the ice-shelf surface are calculated by assuming that the temperature of all shelf ice is equal to Larsen C’s annually averaged surface temperature of −15 °C (Supplementary Fig. 2). Stresses will be higher in colder meteoric ice than in warmer marine ice at a given strain rate, so that marine ice is less likely to fracture^20^,^21^,^23^. The assumption of a spatially invariant temperature of −15 °C is consequently violated in major rift zones filled with warmer ice mélange, where strain rates are therefore anomalously high and stress magnitudes overestimated by our calculations (Supplementary Fig. 2). The assumption is also violated in the suture zones containing warm bodies of marine ice, although here shear stress bridging between neighbouring meteoric flow units minimizes the build-up of anomalously high strain rates, so that stress magnitudes are not normally overestimated. Despite these potential limitations, a comparison of Fig. 2a and Supplementary Fig. 2 reveals good overall agreement between the spatial patterns of principal stresses and stress-flow angles reconstructed from modelled and observed data. However, the patterns inferred from observed data (Supplementary Fig. 2) are relatively noisy and lack prognostic capabilities^4^. We therefore prefer to adopt our previous approach^4^ that focuses on analyses of ice-shelf flow velocities modelled with a continuum-mechanical model constrained by spatially uniform ice properties (Fig. 2).

## Discussion

**Figure 5:**
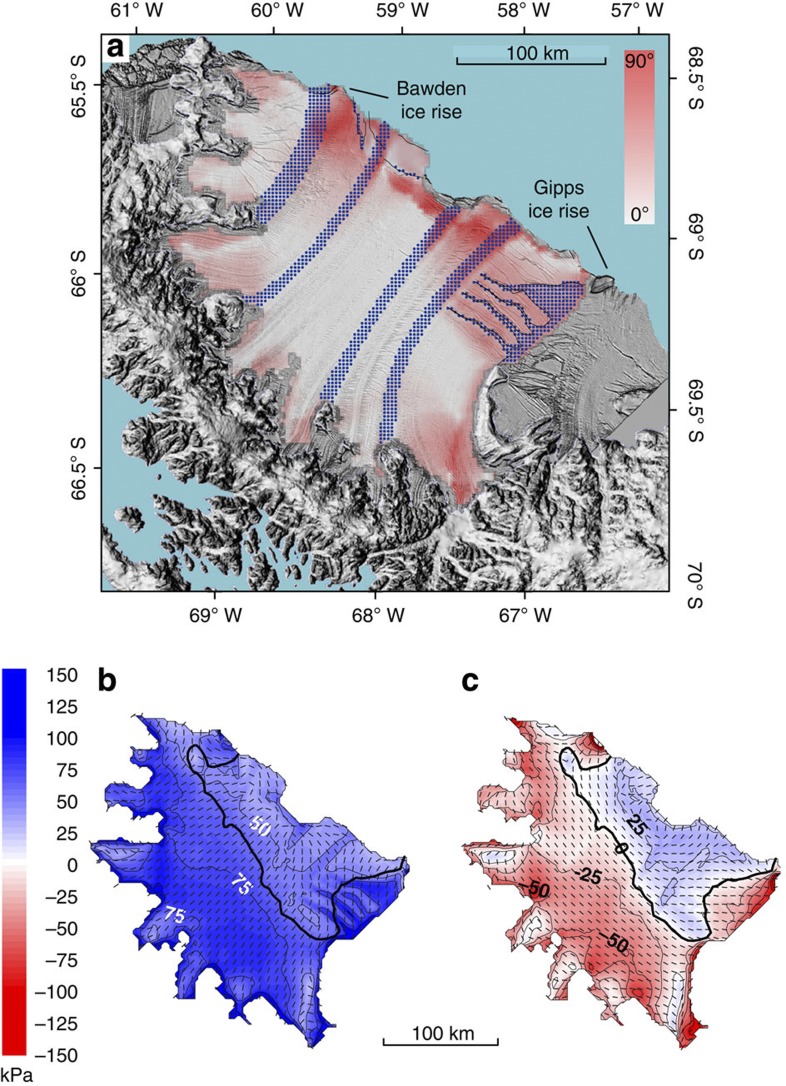
Principal stress fields and stress-flow angles with simulated marine-ice inclusions. Aster-GDEM-derived DEM ( http://nsidc.org/data/docs/agdc/nsidc0516-cook/) is superposed on a 2008 MODIS image ( https://earthdata.nasa.gov/data/near-real-time-data/rapid-response) of Larsen C ice shelf. (**a**) Stress-flow angle distribution. Part of the Churchill Peninsula-derived suture zone is obscured by clouds. Blue hatched zones indicate the suture zones and major rifts implemented for the purpose of perturbation experiments with spatially variable ice rigidity. (**b**) Magnitude (colours) and direction (dashes) of first principal stresses. (**c**) Magnitude (colours) and direction (dashes) of second principal stresses. In **b** and **c**, thick solid lines mark the compressive arch.

Our model sensitivity experiments with simulated softening of marine ice-bearing suture zones and major rifts on Larsen C ice shelf (Fig. 5) reveal that concurrent changes in strain rates within and adjacent to the suture zones act to rotate the trajectories of the first and second principal stresses, respectively, into flow-orthogonal and flow-parallel directions. Inferred stress rotations have two important consequences. First, higher stress-flow angles tend to be focused within marine ice-bearing suture zones, highlighting their rift-stabilizing potential (Fig. 5). Second, the simulated temperature enhancements within suture and rift zones combine to push the compressive arch landwards, especially in the ice-shelf’s northeastern sector, which reduces the likelihood of ice-shelf retreat beyond a critical compressive arch. Together, these two consequences demonstrate that marine ice-bearing suture zones have a critical role in preventing the loss of Larsen C’s frontal portion.

Suture zones on Larsen C ice shelf tend to become thicker and narrower as they are advected downstream, owing to lateral compaction and the balance between continued accretion and basal melting^2^, as demonstrated by our measurements from the Joerg Peninsula suture zone (Fig. 4). Climatically controlled future ocean warming may accelerate upstream marine ice accretion^2^,^24^,^25^, but simultaneously speed-up downstream melting. Melt-induced size reduction of marine ice bodies would decrease their relative influence within a given vertical ice column, reducing that column’s ability to resist fracture^26^. Oceanographic modelling is consistent with the presence of two prominent marine ice-bearing suture zones within Larsen B ice shelf before its collapse, originating downflow of Foyn Peninsula and Cape Disappointment^2^. In the absence of direct observations, we speculate accordingly that these zones were either too thin to resist rift propagation or had been weakened over time due to prolonged basal melting. Larsen C’s northeastern calving front is placed into specific focus because the stress-flow angles in the northeast already approach zero (Fig. 2a). Here the ice shelf is pinned on the Bawden ice rise, stabilizing the large Churchill Peninsula suture zone and rift zones R4 and R5 (Figs 1 and 2a). Retreat from this ice rise due to suture- and rift-zone weakening would cause Larsen C’s northeastern sector to accelerate^27^, and the areal extent of near-zero stress-flow angles to expand towards the south (compare Fig. 2 and Supplementary Fig. 3). Because these consequences would contribute to the de-stabilization of the ice-shelf’s frontal portion, future work should identify the role of the ocean in marine ice accretion and in promoting basal melting^13^,^28^,^29^.

The presence of variably sized bodies of marine ice has already been inferred for several other Antarctic ice shelves, including the three largest, Filchner-Ronne^30^, Ross^31^ and Amery^18^,^25^. Suture zones within these shelves are also readily identifiable from satellite imagery. Our findings thus highlight the need to map marine ice-bearing suture zones within, determine the oceanographic conditions beneath, and model the evolving stress regimes of the many Antarctic ice shelves currently experiencing enhanced erosion by ocean warming. A fundamental question that remains as yet unanswered is why Larsen B’s entire frontal portion calved away in 1995, presenting the initial trigger for its demise. We recommend therefore that future work should also focus on monitoring the evolution of Antarctic ice shelves’ calving fronts in relation to indicative changes in ice flow velocities and crevasse growths. Less feasible at present because of a lack of readily deployed observational techniques, but particularly diagnostic, would be the monitoring of associated changes in the geometry of marine ice inclusions in the shelves’ suture zones. Ultimately marine ice-bearing suture zones contribute to regulating Antarctica’s future contribution to sea-level rise and must therefore be parameterized in ice-sheet models.

## Methods

### Model description

Our continuum-mechanical ice-shelf model^32^ is implemented numerically using a finite-difference scheme with 1.25 × 1.25 km grid cells. The model accounts for gravitational driving forces and associated mechanical stresses, and is based on the shallow-shelf approximation. As such it assumes hydrostatic equilibrium and depth-invariant horizontal flow velocities, and neglects friction at the ice-ocean interface and vertical shear-strain changes because of bending forces. As input, the model requires specification of three-dimensional ice-shelf geometry and the temperature-dependent flow-rate factor B, as well as the inflow velocities at the grounding line boundary of the model domain. The model equations are solved numerically using a force-balance approach^32^, generating as outputs ice-shelf flow velocities and the two-dimensional stress field that is subsequently projected into the principal directions. We previously used the same model implementation to assert the present stability of the Larsen C ice shelf based on the shelf’s geometry in 2002 (ref. 4) before a large tabular calving event (Fig. 1). All model runs reported here are previously unpublished, and focus on Larsen C’s up-to-date post-calving geometry in 2008 (Figs 1 and 2). The 1.25 × 1.25 km size of our grid cells is much larger than the width of individual fractures, but smaller than the areal extent of most major fracture zones.

### Model implementation of marine ice

Our perturbation experiments implemented the most prominent marine ice-bearing suture zones and rifts on Larsen C ice shelf for the purpose of sensitivity testing (Fig. 5). Adopting previous work^4^, we assumed that meteoric ice units have a mean temperature of −12 °C, increasing from −15 °C at the ice-shelf surface to −2 °C at the base. Our implementation assumes a relatively elevated mean suture-zone ice temperature of −7 °C. Here, marine ice with a temperature of −2 °C represents the bottom half of total suture-zone thickness (Fig. 4a), and follows the meteoric ice temperature profile in the suture zone’s top half. These values of meteoric and marine ice temperatures are vertically averaged to yield the flow-rate factor B for any given vertical ice column in our 1.25 × 1.25 km model grid. Our implementation further assumes that the rifts in Larsen C’s south-eastern sector (Fig. 5) are filled with an ice mélange^24^ that has a vertically constant temperature of −2 °C between the ice-shelf surface and its base.

### Acquisition and processing of GPR data

The GPR data presented in Fig. 4 were acquired during the austral summers of 2008–09 (ref. 4) and 2009–10 (ref. 5) using a high-power Sensors and Software PE-Pro (2008–09) or older and lower-power PE-100 (2009–10) GPR systems with 50 MHz antennas, operated in common offset mode using a snow-scooter-towed sledge assembly. Choice of the 50 MHz antennas provided the best compromise between depth penetration and vertical resolution, following a suite of testing using several antennas of different frequencies in 2008–09. The GPR data were acquired with a 0.8-ns sampling interval, where each trace was acquired eight times and then stacked into a single recorded trace. A compromise between towing speed as well as required window length and number of stacks for each trace implied that one trace was recorded ~every 3–4 m along the survey lines. In 2008–09, a handheld GPS was linked to the GPR system, locating each individual recorded radar trace with a planimetric precision of approximately±5 m. In 2009–10, precise (±0.1 m) planimetric and height location of the antennas was achieved with a differential Leica System 1200 GPS. The raw radar data were processed using the commercial Reflex-W package. Standard techniques were applied, including de-wow, automatic gain control and band-pass filter, as well as adjustment for noticeable surface topography in 2009–10 immediately downstream of the Joerg Peninsula (Figs 1 and 4). The data are presented as un-migrated profiles because the meteoric-marine ice interfaces are more readily distinguishable in this format. Travel times were converted to depth assuming a depth-averaged radar velocity of 0.175 m ns^−1^ based on common-midpoint surveys conducted in both field seasons^4^,^5^. The use of the older and lower-power PE-100 GPR system in 2009–10 resulted in much noisier data (Fig. 4b) than those collected in 2008–09 (Fig. 4a), although marine ice bodies are still readily distinguishable (Fig. 4b).

### Determination of suture-zone thicknesses

Our seismic reflection data were acquired in walk-away mode in 2008–09 (ref. 4) using a Geometrics-Geode-based system and explosive shots deployed in shallow drill holes, yielding an estimate of the suture-zone thickness at P1 (Figs 1 and 4a). No seismic data were available in 2009–10. Instead the suture-zone thickness at P2 (Figs 1 and 4b) was calculated using (a) the precise differential GPS data available for P2, under the assumption of hydrostatic equilibrium and a mean density profile derived from the 2008/09 seismic data^4^; and (b) a recently published method for the estimation of firn-air content on Larsen C^12^. Results from both methods agree to within 0.5%.

## Author contributions

B.K. and A.J.L. respectively led NERC projects NE/E012914/1 and NE/I016678/1 that made this work possible; and D.J. was the post-doctoral scientist for both. E.C.K. and P.R.S. were co-investigators on NE/E012914/1. D.J. implemented and conducted the ice-shelf model runs presented here. Building on E.C.K.’s expertise, B.K. and A.J.L. collected the field geophysical data used as model constraints; and B.K., E.C.K. and D.J. processed them. A.J.L. and D.J. acquired and processed the satellite data used as model constraints. P.R.S. contributed on the mechanical properties of and fracture processes operating on ice shelves.

## Additional information

**How to cite this article:** Kulessa, B. *et al.* Marine ice regulates the future stability of a large Antarctic ice shelf. *Nat. Commun.* 5:3707 doi: 10.1038/ncomms4707 (2014).

## Supplementary Material

Supplementary Information Supplementary Figures 1-3

## References

[b1] Vieli A., Payne A. J., Shepherd A. & Du Z. Causes of precollapse changes of the Larsen B ice shelf: numerical modelling and assimilation of satellite observations. Earth Planet. Sci. Lett. 259, 297–306 (2007).

[b2] Holland P. R., Corr H. F. J., Vaughan D. G., Jenkins A. & Skvarca P. Marine ice in Larsen Ice Shelf. Geophys. Res. Lett. 36, L11604 (2009).

[b3] Glasser N. F. *et al.* Surface structure and stability of the Larsen C ice shelf, Antarctic Peninsula. J. Glaciol. 55, 400–410 (2009).

[b4] Jansen D. *et al.* Present stability of the Larsen C ice shelf, Antarctic Peninsula. J. Glaciol 56, 593–600 (2010).

[b5] Luckman A. *et al.* Basal crevasses in Larsen C ice shelf and implications for their global abundance. The Cryosphere 6, 113–123 (2012).

[b6] McGrath D. *et al.* Basal crevasses on the Larsen C ice shelf, Antarctica: Implications for meltwater ponding and hydrofracture. Geophys. Res. Lett. 39, L16504 (2012).

[b7] McGrath D. *et al.* Basal crevasses and associated surface crevassing on the Larsen C ice shelf, Antarctica, and their role in ice-shelf instability. Ann. Glaciol. 53, 10–18 (2012).

[b8] Doake C. S. M. *et al.* Breakup and conditions for stability of the northern Larsen Ice Shelf, Antarctica. Nature 391, 778–780 (1998).

[b9] Borstad C. P. *et al.* A damage mechanics assessment of the Larsen B ice shelf prior to collapse: Toward a physically-based calving law. Geophys. Res. Lett. 39, L18502 (2012).

[b10] Rack W. & Rott H. Pattern of retreat and disintegration of Larsen B ice shelf, Antarctic Peninsula. Ann. Glaciol. 39, 505–510 (2004).

[b11] Khazendar A., Rignot E. & Larour E. Acceleration and spatial rheology of Larsen C ice shelf, Antarctic Peninsula. Geophys. Res. Lett. 38, L09502 (2011).

[b12] Holland P. R. *et al.* The air content of Larsen Ice Shelf. Geophys. Res. Lett. 38, L10503 (2011).

[b13] Pritchard H. D. *et al.* Antarctic ice-sheet loss driven by basal melting of ice shelves. Nature 484, 502–505 (2012). 22538614 10.1038/nature10968

[b14] Fricker H. A. & Padman L. Thirty years of elevation change on Antarctic Peninsula ice shelves from multimission satellite radar altimetry. J. Geophys. Res. 117, C02026 (2012).

[b15] Pritchard H. D., Arthern R. J., Vaughan D. G. & Edwards L. A. Extensive dynamic thinning on the margins of the Greenland and Antarctic ice sheets. Nature 461, 971–975 (2009). 19776741 10.1038/nature08471

[b16] Shepherd A., Wingham D., Payne T. & Skvarca P. Larsen Ice Shelf has progressively thinned. Science 302, 856–859 (2003). 14593176 10.1126/science.1089768

[b17] Dierckx M. & Tison J.-L. Marine ice deformation experiments: an empirical validation of creep parameters. Geophys. Res. Lett. 40, 134–138 (2013).

[b18] Craven M., Allison I., Fricker H. A. & Warner R. Properties of a marine ice layer under the Amery Ice Shelf, East Antarctica. J. Glaciol. 55, 717–728 (2009).

[b19] Jansen D., Luckman A., Kulessa B., Holland P. R. & King E. C. Marine ice formation in a suture zone on the Larsen C ice shelf and its influence on ice shelf dynamics. J. Geophys. Res. Earth Surf. 118, 1–13 (2013).

[b20] Rist M. A., Sammonds P. R., Oerter H. & Doake C. S. M. Fracture of Antarctic shelf ice. J. Geophys. Res. 107, 2002 (2002).

[b21] Vaughan D. G. Relating the occurrence of crevasses to surface strain rates. J. Glaciol. 39, 255–266 (1993).

[b22] Rignot E., Mouginot J. & Scheuchl B. MEaSUREs InSAR-Based Antarctica Ice Velocity Map. Science 333, 1427–1430 (2011). 21852457 10.1126/science.1208336

[b23] Rist M. A. *et al.* Experimental and theoretical fracture mechanics applied to Antarctic ice fracture and surface crevassing. J. Geophys. Res. 104, 2973–2987 (1999).

[b24] Khazendar A. & Jenkins A. A model of marine ice formation within Antarctic ice shelf rifts. J. Geophys. Res. 108, 3235 (2003).

[b25] Galton-Fenzi B. K., Hunter J. R., Coleman R., Marsland S. J. & Warner R. C. Modeling the basal melting and marine ice accretion of the Amery Ice Shelf. J. Geophys. Res. 117, C09031 (2012).

[b26] McGrath D. *et al.* The structure and effect of suture zones in the Larsen C ice shelf, Antarctica. J. Geophys. Res. Earth Surf. 119, doi:10.1002/2013jf002935 (2014).

[b27] Borstad C. P., Rignot E., Mouginot J. & Schodlok M. P. Creep deformation and buttressing capacity of damaged ice shelves: theory and application to Larsen C ice shelf. The Cryosphere 7, 1931–1947 (2013).

[b28] King M. A., Makinson K. & Gudmundsson G. H. Nonlinear interaction between ocean tides and the Larsen C ice shelf system. Geophys. Res. Lett. 38, L08501 (2011).

[b29] Hellmer H. H., Kauker F., Timmermann R., Determann J. & Rae J. Twenty-first-century warming of a large Antarctic ice-shelf cavity by a redirected coastal current. Nature 485, 225–228 (2012). 22575964 10.1038/nature11064

[b30] Lambrecht A., Sandhäger H., Vaughan D. G. & Mayer C. New ice thickness maps of Filchner–Ronne Ice Shelf, Antarctica, with specific focus on grounding lines and marine ice. Antarct. Sci. 19, 521–532 (2007).

[b31] Rommelaere V. & MacAyeal D. Large-scale rheology of the Ross Ice Shelf, Antarctica, computed by a control method. Ann. Glaciol. 24, 43–48 (1997).

[b32] Grosfeld K. & Sandhäger H. The evolution of a coupled ice shelf–ocean system under different climate states. Global Planet. Change 42, 107–132 (2004).

