# Supplementary material for: Marine ice regulates the future stability of a large Antarctic ice shelf
Source: Nat Commun. 2014 Apr 22;5:3707. doi: 10.1038/ncomms4707 (PMC3997805; doi:10.1038/ncomms4707)
Supplement: Supplementary Information — Supplementary Figures 1-3 [file ncomms4707-s1.pdf]

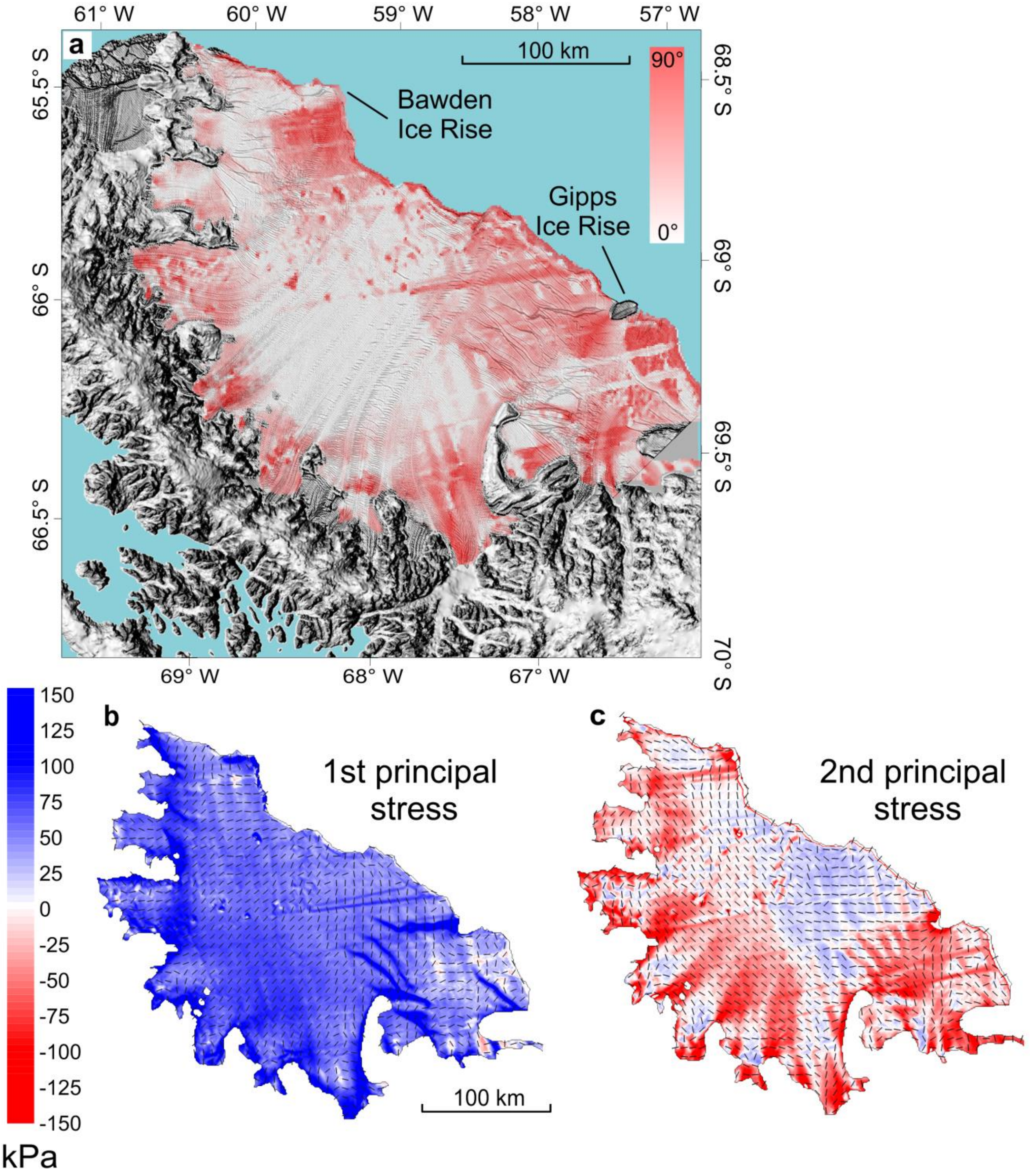

Supplementary Figure 1 | Principal stress fields and stress-flow angles calculated from observed velocity data. Aster-GDEM derived DEM [http://nsidc.org/data/docs/agdc/nsidc0516-cook/] is superposed on a 2008 MODIS Mosaic of Antarctica (MOA) image of Larsen C ice shelf [https://earthdata.nasa.gov/data/near-real-time-data/rapid-response]. (**a**) Stress-flow angle distribution. Part of the Churchill-Peninsula derived suture zone is obscured by clouds. (**b**) Magnitude (colours) and direction (grey dashes) of first principal stresses. (**c**) Magnitude (colours) and direction (grey dashes) of second principal stresses.

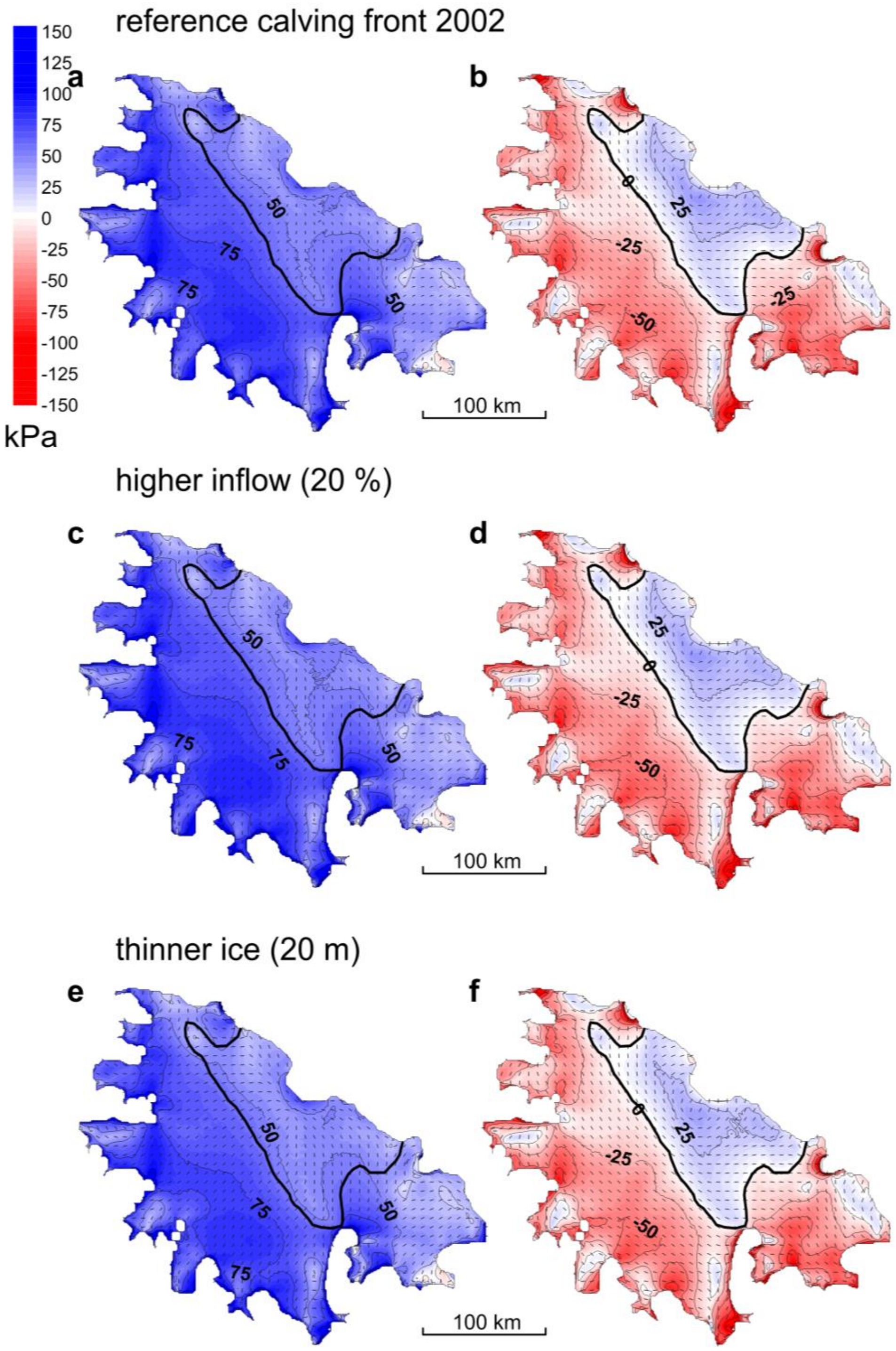

Supplementary Figure 2 | Principal stress fields for three representative model sensitivity experiments. All images use Larsen C's 2002 geometry prior to a large tabular calving event; compare with 2008 post-calving results in Fig. 2 in main article. Colours and grey dashes represent respectively stress magnitudes and directions, and compressive arches are marked by thick solid lines. (**a**) First principal stresses. (**b**) Second principal stresses. (**c**) First principal stresses for 20% higher inflow velocities of the feeding glaciers. (**d**) Second principal stresses for 20% higher inflow velocities of the feeding glaciers. (**e**) First principal stresses for a Larsen C ice shelf thinner by 20m. (**f**) Second principal stresses for a Larsen C ice shelf thinner by 20m.

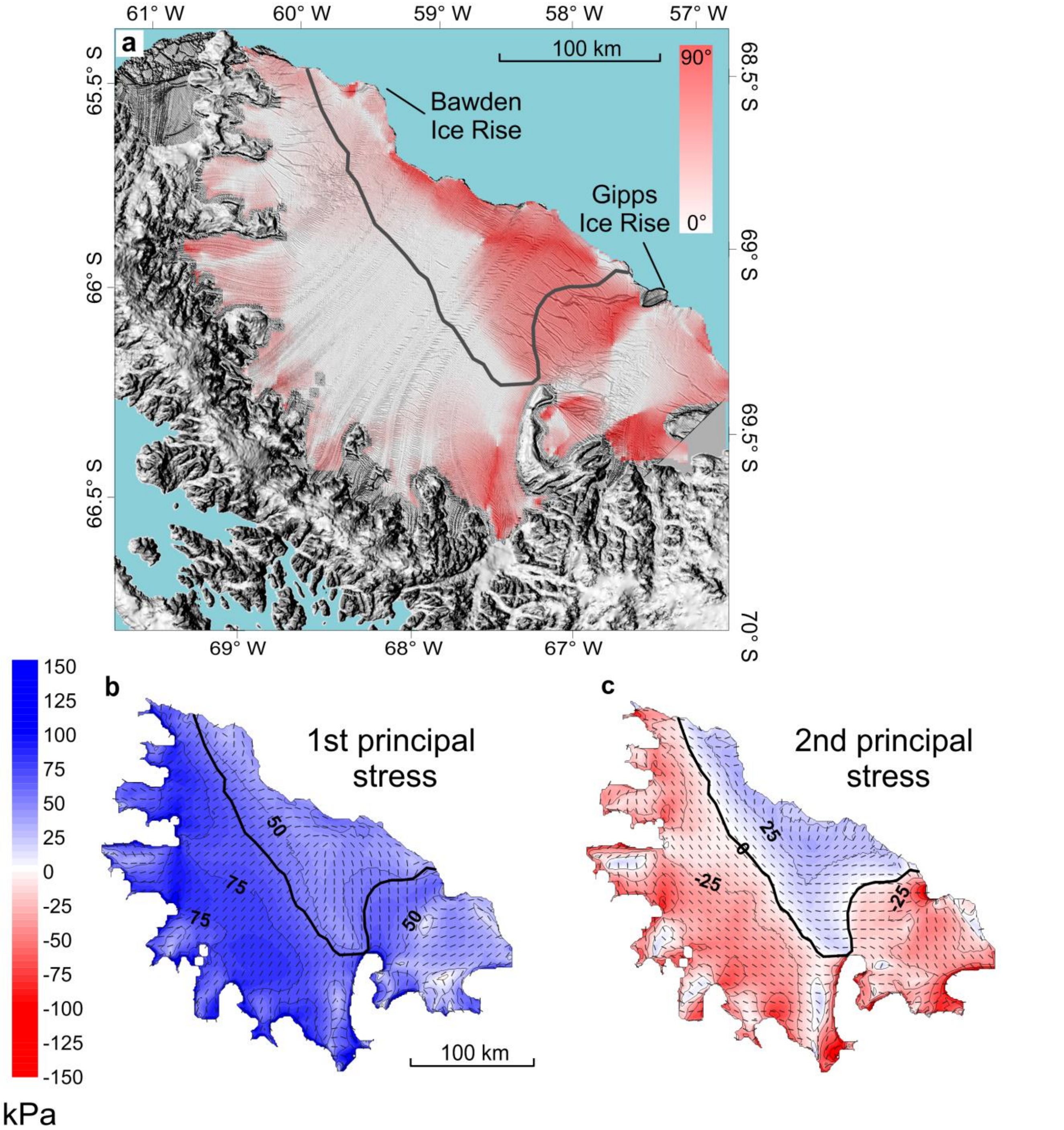

Supplementary Figure 3 | Principal stress fields and stress-flow angles for a hypothetical retreat from Bawden ice rise. Calculations use modelled flow-velocity data for Larsen C ice shelf. Aster-GDEM derived DEM [http://nsidc.org/data/docs/agdc/nsidc0516-cook/] is superposed on a 2008 MODIS image [https://earthdata.nasa.gov/data/near-real-time-data/rapid-response]. Compare with Fig. 2 in main article. Thick solid lines mark the location of the compressive arch. **a**, Stress-flow angle distribution. Part of the Churchill-Peninsula derived suture zone is obscured by clouds. The solid grey lines indicate the compressive arch. **b**, Magnitude (colours) and direction (grey dashes) of first principal stresses. **c**, Magnitude (colours) and direction (grey dashes) of second principal stresses.
